# Functional androdioecy in the ornamental shrub *Osmanthus delavayi* (Oleaceae)

**DOI:** 10.1371/journal.pone.0221898

**Published:** 2019-09-05

**Authors:** Yifan Duan, Weihong Li, Sunyuan Zheng, Steven Paul Sylvester, Yongfu Li, Fuyue Cai, Cheng Zhang, Xianrong Wang

**Affiliations:** 1 Co-Innovation Center for Sustainable Forestry in Southern China, Nanjing Forestry University and International Cultivar Registration Center for *Osmanthus*, Nanjing, Jiangsu, P. R. China; 2 Department of Plant Science, College of Biology and the Environment, Nanjing Forestry University, Nanjing, Jiangsu, P. R. China; 3 Nanjing Foreign Language School, Nanjing, Jiangsu, P. R. China; United States Department of Agriculture, UNITED STATES

## Abstract

Androdioecy is one of the rarest sexual systems among plants, characterized by males co-occurring with hermaphrodites. *Osmanthus delavayi* (Oleaceae), an ornamental shrub from southern China, is known to have both male and hermaphrodite individuals, but little is known regarding the breeding system of this species and whether it is functionally androdioecious, and how this potentially evolved. In this study, we explore the characteristics of the breeding system of *O*. *delavayi* through the study of phenology, sex ratio, floral organ morphology, pollen number, stigma receptivity, artificial pollination, pollinators, and gene flow within and between populations, while also discussing the evolution and maintenance of androdioecy within the genus. The proportion of males was less than 0.5 and the out-crossing index (OCI) was 5. Morphological androdioecy was observed, with hermaphrodite flowers having fertile pistils, while male flowers had degenerated pistils. Males and hermaphrodites both had large amounts of small and fertile pollen grains, although the pollen number of males was ca. 1.21 × more than that of hermaphrodites, and pollen was generally smaller. Self-pollination was found to produce a much lower fruit set than outcrossing under natural conditions. Gene flow between males and hermaphrodites within a population was greater (1.007) than that between populations (0.753). All these results indicate that *O*. *delavayi* is functionally androdioecious, which may be an intermediate state in the evolutionary transition from hermaphroditism to dioecy.

## Introduction

Androdioecy is a rare (<0.005%), but phylogenetically important, breeding system in angiosperm species [1, 2] that is characterized by populations containing both male and hermaphroditic individuals. Some morphological androdioecy has, however, been shown to be cryptic dioecy [3, 4] with a sex ratio of 1:1 as the hermaphrodites express only female function [5]. Theory suggests that, to preserve androdioecy, male fitness must be at least twice as high as that of the male function in hermaphrodites [5, 6]. In some cases, higher frequencies of males in populations of androdioecious plants has been explained by how, in the species studied, hermaphrodites belong to one of two self-incompatible groups and so males have an advantage as they are fully compatible with all pollen recipients while hermaphrodites can only fertilize pollen recipients from the opposite self-incompatible group [7, 8]. High clonal reproduction in males has also been shown to increase male pollen volume and fitness [9]. Androdioecy is hypothesized to have evolved during the transition from hermaphrodism to dioecy by the invasion of a female-sterile mutant [10–13]. Barrett proposed that an evolutionary reversal from dioecy to androdioecy is implausible because of pollen limitation [14, 15], and this has been corroborated by phylogenetic and morphological evidence from certain androdioecious herbaceous plant groups [16–18].

Although generally rare, androdioecy is more common in annual herbaceous plants and the olive family, Oleaceae [19], with more than 37 species in Oleaceae exhibiting androdioecy. Most of these species belong to the genera *Fraxinus* L., *Phillyrea* L., and *Chionanthus* L., with several species, e.g. *Fraxinus longicuspis* Siebold et Zucc. [20] and *Chionanthus retusus* Lindl. et Paxt. [21], already confirmed to be androdioecious. The genus *Osmanthus* Lour. has received very little study regarding its breeding system, with only *Osmanthus fragrans* (Thunb.) Lour. [22], the type species of *Osmanthus* with a cultivation history of over 2500 years [23] in China, confirmed as androdioecious [24]. *Osmanthus* comprises ca. 35 species distributed in North America, New Caledonia and East Asia, mainly in China [25, 26], with it likely that many of these species are androdioecious.

**Fig 1:**
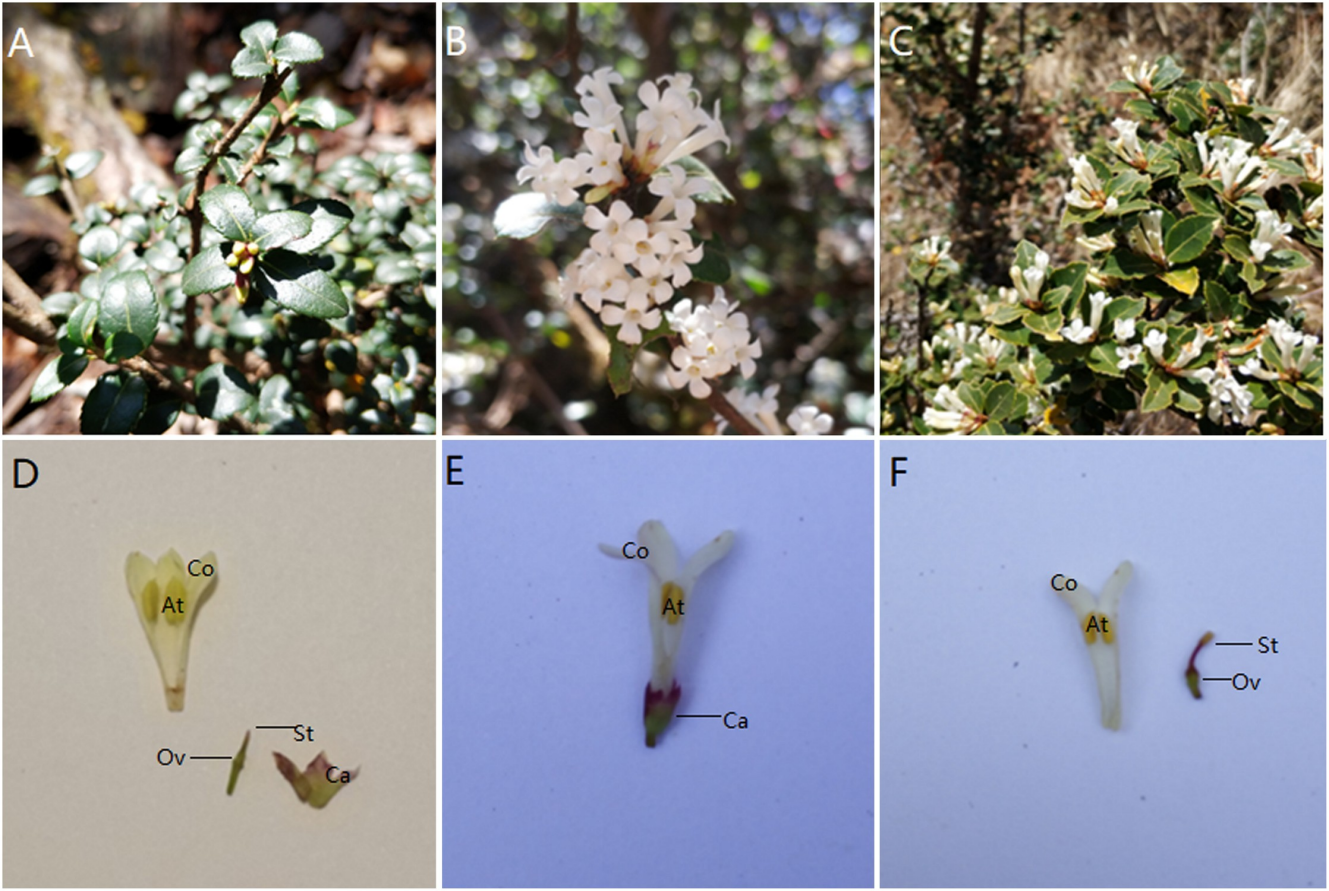
*Osmanthus delavayi* male and hermaphrodite inflorescence characteristics. Note: (A) Habit of male. (B) Inflorescence of male. (C) Inflorescence of hermaphrodite. (D) Structure of male flower. (E, F) Structure of hermaphrodite flower (At: anther, Ca: calyx, Co: corolla, Ov: ovary, St: stigma.).

*Osmanthus delavayi* Franch. (*Osmanthus* Sect. *Siphosmanthus* Franch.) is an endemic spring-flowering species native to the Guizhou, Sichuan and Yunnan regions of southern China [27]. It is an evergreen shrub with opposite, oval or ovate, serrated leaves and white tubular flowers (Fig 1A–1C). It exhibits morphological androdioecy, with expanded stigmas in hermaphrodites and degraded pistils in males [26]. Here, we studied various characteristics of the breeding system to clarify if *O*. *delavayi* is functionally androdioecious, while also discussing how this breeding system possibly evolved and potential future lines of research, which will benefit our understanding of the evolution of breeding systems in plants, in general, and, in particular, that of the genus *Osmanthus*.

## Materials and methods

### Material and location

Study materials were collected during 2016–2018 from six populations. One population (referred to here as ‘CS’) was located on the west slope of Cang Mountain (25°47’33”N; 99°59’24”E; 3058 m) in Yangjiang Town, Yangbi County, west of Dali Yi Autonomous Prefecture. The other five populations (JZ1-5) were located on Jizu Mountain in Binchuan County (25°58’N; 100°21’E; 2814–3250 m), east of Dali Yi Autonomous Prefecture. Our research does not involve endangered or protected species. There is no special permit for all study areas. Because the Jizu Mountain is just a famous Buddhist mountain in Yunnan, and the west slope of Cang Mountain has not been developed into a tourist place. Locations are all publicly owned.

### Sex ratio and floral traits

We sampled one 20 m × 20 m plot in each of the six populations and calculated male ratio within each plot. To identify the morphological differences of male and hermaphrodite flowers, we then measured floral traits of 20 flowers per tree of 20 individual trees in each plot using an electronic digital vernier caliper. The out-crossing index (OCI) index was calculated to assess the breeding system of *O*. *delavayi* [28]. Flowers were collected and preserved in FAA (38% formalin: acetic acid: 70% ethyl alcohol = 1:1:18) and then dehydrated using gradient alcohol and made gradually transparent in xylene, following which they were embedded in melted paraffin at 60 °C (BINDEA, FED53, Germany). 8 μm longitudinal sections were cut by routine paraffin sectioning. After safranin-fast green FCF staining, the sections were dried and subsequently observed and photographed under an optical microscope (Nikon upright microscope CI-S, Japan) to investigate the floral structure.

### Examination of pollen characteristics, pollen-ovule ratio, and pollen vitality

For measurement of pollen number, anthers of male and hermaphrodite flowers from 10 individuals per population were individually broken up and the volume was adjusted to 1.5 ml with 10% hydrochloric acid. We took 2 microliters out each time and counted pollen numbers from 5 fields under an optical microscope, repeated these 10 times to get the average data. Ovaries of hermaphrodites were dissected under stereoscope (Nikon SMZ745T, Japan) to count ovule number, and the pollen-ovule (P/O) ratio was then calculated based on the pollen number obtained, which in turn allowed the breeding system of *O*. *delavayi* to be assessed according to the criteria of Cruden [29]. Anthers of male and hermaphrodite flowers were dehydrated by gradient alcohol dehydration and then immersed in carbon dioxide for critical point drying. Anthers were attached to a metal plate and pricked carefully to make the dried pollen grains disperse. The metal plate was then placed into an ion sputter device and coated with platinum. An environmental scanning electron microscope (E-SEM, FEI, Quanta200, America) was utilized to observe and identify the pollen. The dimensions of 40 pollen grains from both male and hermaphrodite flowers were calculated using Macni-fication version 2.0 software (Orbicule, Leuven, Belgium).

Artificial pollination experiments were used to measure pollen vitality. Pollen was collected from 20 male anthers and 20 hermaphrodite anthers, and then separately applied on the heads of 40 hermaphrodite stigmas with a soft brush. After pollination, stigmas were labelled and sealed in polyester bags to prevent contamination. After 12 h, 24 h and 48 h, stigmas were removed and preserved in FAA. For staining, stigmas were then rinsed with distilled water, softened with 0.5 mol/L sodium hydroxide, washed with plenty of potassium metaborate buffer, stained with 0.01% water-soluble aniline blue for 20 minutes, and finally sealed with 50% glycerin. The germination and growth of pollen tubes from male and hermaphrodite flowers were then observed and compared under a fluorescent microscope (Nikon eclipse upright microscope ci-s, Japan).

### Stigma receptivity tests and pollinators

Stigma receptivity was examined at five stages during the flowering period: (1) Bud stage: Inflorescence has spread but petals have not expanded; (2) Near-flowering stage: Petals spirally open; (3) Early-flowering stage: About 10% of flowers are blooming; (4) Peak-flowering stage: 70% of flowers are blooming, anthers begin to release pollen; (5) Final-flowering stage: Petals begin to wither and fall off. Ten stigmas from each flowering stage were collected and placed on their concave side in benzidine-hydrogen peroxide reaction solution (1% benzidine:3% hydrogen peroxide: water = 4:11:22). Receptive stigmas would turn blue and produced a lot of bubbles, if not receptive they remained unchanged.

The study of pollinators was conducted in two populations, JZ4 and JZ5, on the top of Jizu Mountain. Flowers were observed for 1 hour at 8:00am, 11:00am, 2:00pm and 5:00pm, respectively, over 3 days from 20th March to 22th March 2018, and insects were captured to record the visitors and visiting frequency.

### Mating system

#### Artificial pollination tests

We set up nine types of artificial pollination test in populations JZ4 and JZ5 of JiZu Mountain, with tests 1–8 focused on flowers of hermaphrodite trees and test 9 focused on flowers of male trees: (1) Natural: stamens preserved in flowers and flowers not sealed in bags, no hand pollination; (2) stamens preserved in flowers and flowers sealed in bags; (3) stamens removed from flowers, stigmas hand-pollinated with pollen from flowers of the same tree, flowers then sealed in bags; (4) stamens removed from flowers, stigmas hand-pollinated with pollen from flowers of other hermaphrodite trees, flowers not sealed in bags; (5) stamens removed from flowers, stigmas hand pollinated with pollen of male flowers, flowers not sealed in bags; (6) stamens preserved in flowers, flowers covered in gauze to prevent pollinators visiting, no hand pollination, flowers not sealed in bags; (7) stamens removed from flowers, flowers covered in gauze to prevent pollinators visiting, flowers not sealed in bags, no hand pollination; (8) stamens removed from flowers and flowers sealed in bags, no hand pollination; (9) Pollination of degenerated stigmas of male plants by hand, flowers not sealed in bags. In each test, flowers were picked after 24 and 48 hours and preserved in FAA softened in 5 mol/L sodium hydroxide. After washing stigmas with buffer solution, water-soluble aniline blue was used for staining them. Pollen tube germination and ovary fertilization were observed using a fluorescent microscope, allowing ovary fertilization rates of the nine types of pollination test to be calculated.

#### Gene flow

We measured gene flow indirectly using the relationship between gene flow (*Nm*) and *F*_*st*_: *Nm* = 1/(4*Fst*+1) [30]. Leaves of males and hermaphrodites in the six populations were collected and dried with silica gel. 50 mg of dried leaves were quickly frozen and ground into powder in liquid nitrogen. DNA of *O*. *delavayi* was extracted using a DNA extraction kit (TIAN quick midi purification kit), centrifuge (HEXI HR/T18MM Micro high speed refrigerated centrifuge, China) and thermostat water bath (SENXIN, DK-S16, Shanghai). Nine pairs of primers (OSM005、OSM010、OSM011、OSM014、OSM017、OSM023、OSM025、OSM036、0SM052) with high stability and polymorphism were obtained from synthesizing 15 pairs of *O*. *delavayi* fluorescence primers [31]. Amplification products were produced by polymerase chain reaction using a PCR machine (Coyote Bio CF-F9677, America), and were submitted to agarose gel electrophoresis (HDL apparatus DL-EP300) to detect the amplification effect. PCR products were then sent to Qingke Biosciences for capillary electrophoresis. Data analysis was performed on the returned results to calculate gene flow between males and hermaphrodites as well as between populations.

### Statistical analyses

We used two-way analysis of variance (ANOVA) to compare differences in floral traits between males and hermaphrodites and between the six populations. We also carried out an independent t-test and Mann-Whitney U test to analyse the differences in quantitative pollen data between hermaphrodite and male flowers. A Pearson correlation test was performed to study the correlation between pollen characteristics. All analyses were conducted using SPSS statistical software (IBM SPSS Statistics for Windows, Version 19.0., released 2010, IBM Corp., Armonk, USA).

## Results

### Male ratio and floral traits (Reproductive organs’ structure and size)

**Fig 2:**
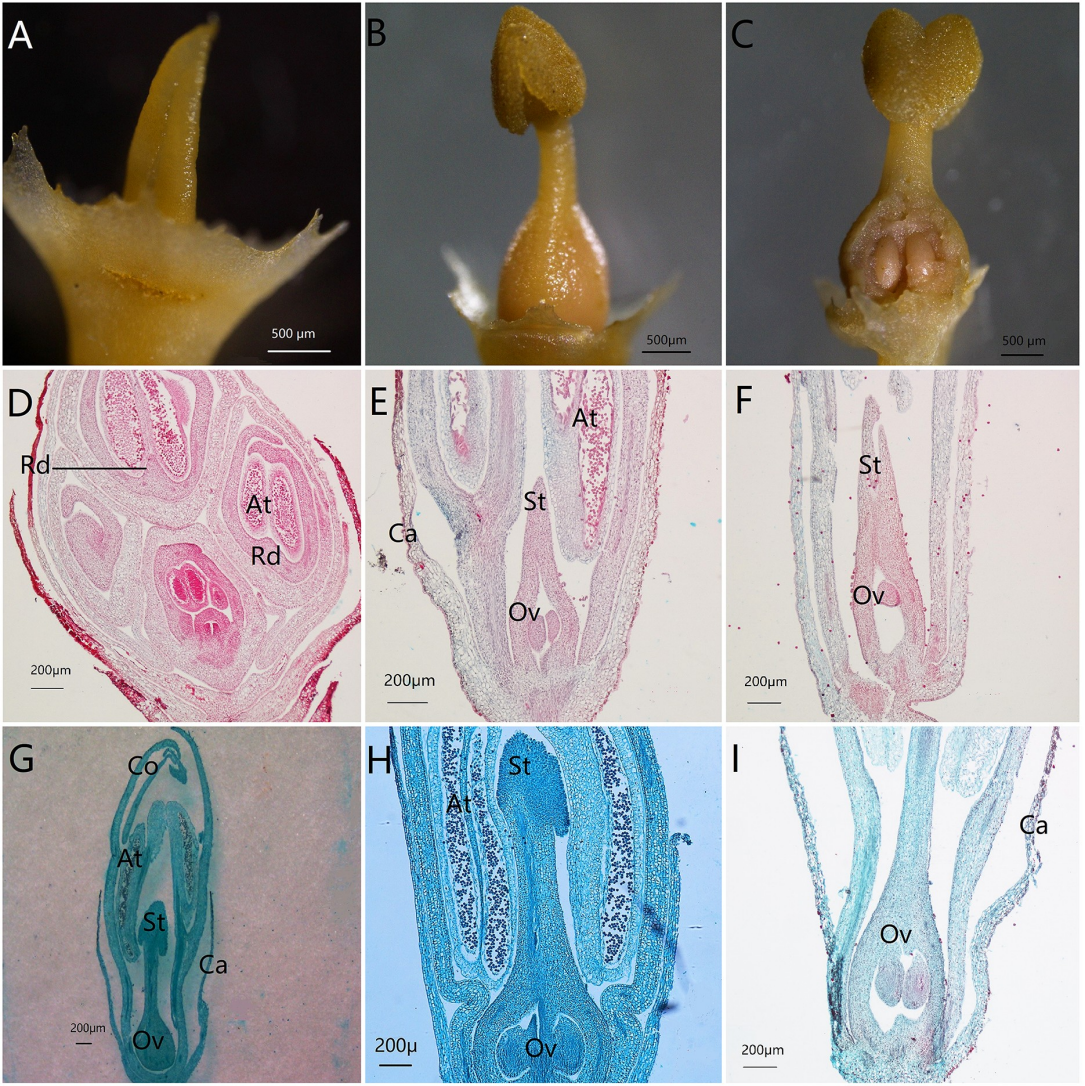
SEM micrographs of two sexual pistils and paraffin section micrographs of floral dimorphism in *O*. *delavayi*. Note: (A, D-F) Male. (B, C, G-I) Hermaphrodite. (A-C) Pistils of the two sexual morphs. (D-I) Longitudinal section of reproductive organs. Abbreviations: At: anther, Ca: calyx, Co: corolla, Ov: ovary, Rd: rudimentary pistil, St: stigma.

**Fig 3:**
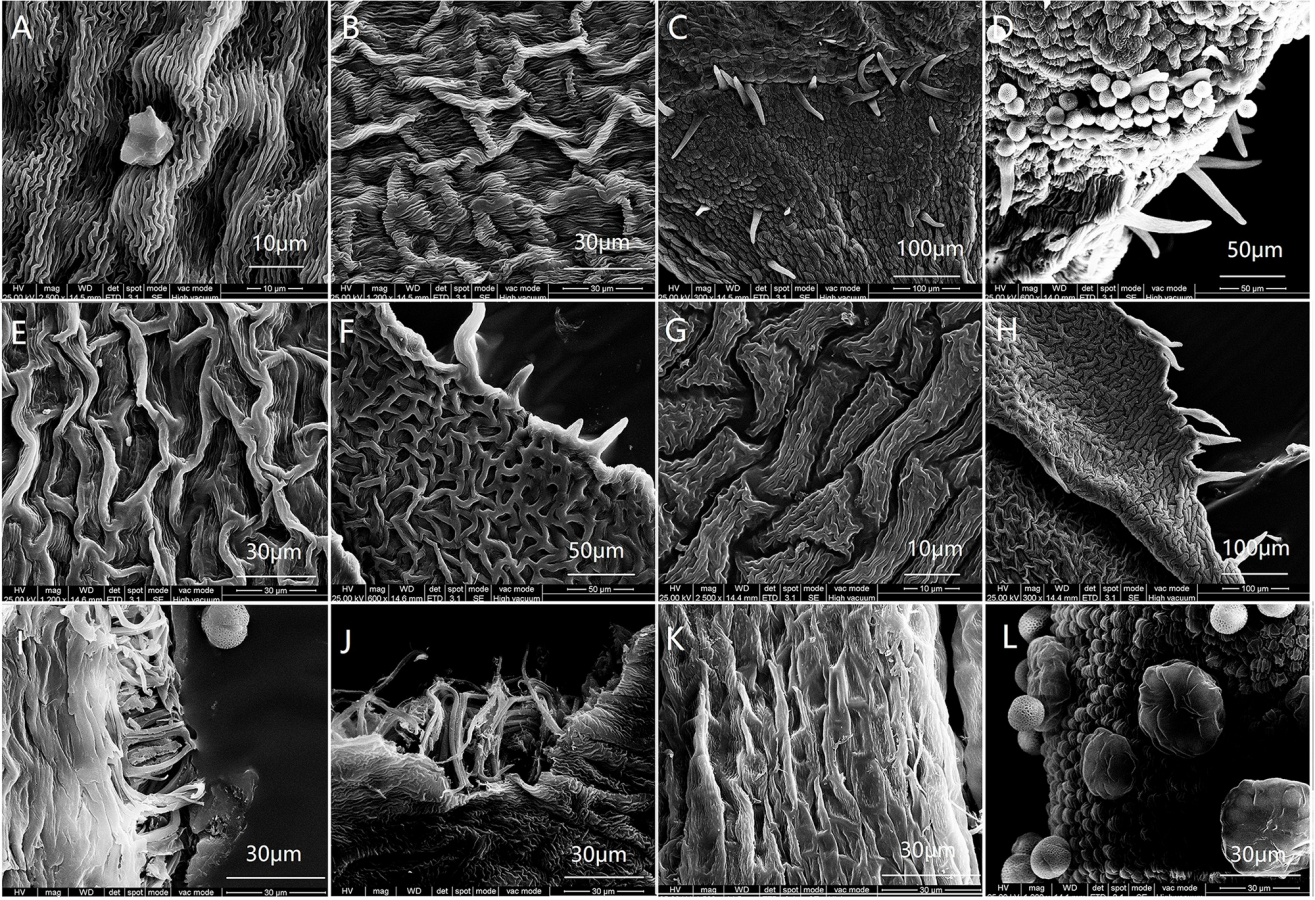
SEM micrographs of floral micromorphology in *O*. *delavayi*. Note: (A, C, E, F, I, K) Male. (B, D, G, H, J, L) Hermaphrodite. (A, B) Inner corolla. (C, D) Torus surface, trichomes present. (E-H) Calyx inner epidermal cells, margin pilose. (I, J) Inner anther, tapetum present. (K) Stigma of male, papillae present. (L) Ovary epidermal cells of hermaphrodite, papillae present.

The proportion of males in each of the 6 populations was below 50% (Table 1). The habit, inflorescence and structure of males (Fig 1A, 1B and 1D) and hermaphrodites (Fig 1C, 1E and 1F) are shown in Fig 1. Morphologically, hermaphrodites had a fully developed ovary with 4 ovules, as well as a long style emerging from the top of the ovary with an enlarged stigma (Figs 1F, 2C and 2D), while the ovary of male flowers was not developed, and the stigma was not enlarged, but rather a small cusp (Figs 1D, 2A and 2B). Photomicrographs of paraffin sections showed that the pistil of male flowers developed from two carpels into one empty chamber during flower bud development (Fig 2E–2G). The pistil of the hermaphrodite flower formed an enlarged stigma and ovary with four oval ovules inside (Fig 2H–2J). SEM data for floral microstructure found that males and hermaphrodites only exhibited a few differences in terms of inner corolla, cell structure of anthers and surface of stigmas (Fig 3). Cells of the inner corolla of males were rarely papillose, while those of hermaphrodites were never papillose (Fig 3A and 3B). Both receptacles had epidermal trichome (Fig 3C and 3D). Both males and hermaphrodites had trichomes present on the calyxes (Fig 3E–3H). Ovaries of hermaphrodites had numerous spheroidal papillae on their epidermis, while ovaries of males lacked papillae (Fig 3K and 3L). A thick tapetum was observed inside the anthers of both males and hermaphrodites (Fig 3I and 3J). Phenological studies found the majority of male flowers in the study flowered 2–3 days earlier than those of hermaphrodites (Male: 18th of April; hermaphrodite: 21th of April). When male flowers bloomed vigorously and anthers cracked, the hermaphrodite flowers were reaching their flowering stage.

**Table 1:** Male ratio of *O*. *delavayi* from 6 populations in Yunnan.

population	Elevation/m	male	hermaphrodite	Total number	male ratio/%
CS	3058	7	16	23	30
JZ1	3207	15	38	53	28
JZ2	3104	43	67	110	39
JZ3	2982	42	65	107	39
JZ4	2908	40	52	92	43
JZ5	2814	20	30	50	40

Two-way ANOVA showed significant differences in the size of the floral organs associated with sexual morph and populations (Tables 2 and 3). Anther length (F = 68.64, P<0.001) of males was 1.31 times longer than that of hermaphrodites. Pistils of hermaphrodites were much larger than that of males in terms of stigma length (1.76 times longer; F = 180.98, P<0.001) and ovary diameter (1.62 times wider; F = 164.90, P<0.001) (Tables 2 and 3). Significant differences were also found in the same sexual morphs (i.e. males or hermaphrodites) among the 6 populations, especially for sepal length (F = 7.76, P<0.001) and corolla width (F = 3.99, P<0.01). The correlation between the sites and the sexual morphs on the floral traits was significant in corolla width (F = 7.39, P<0.001), corolla length (F = 3.93, P<0.01) and anther length (F = 4.45, P<0.01) (Table 3).

**Table 2:** Measurements (Mean± S. E.) of floral traits of males and hermaphrodites of *O*. *delavayi* from 6 populations.

Sex	Populations	Corolla length	Corolla width	Anther length	Anther width	Stigma length	Ovary diameter	Sepal length	Sepal width
Hermaphrodite	CS	12.40±0.35	9.02±1.32	2.46±0.14	0.81±0.08	4.45±0.49	1.18±0.14	3.50±0.41	2.06±0.14
	JZ1	11.90±0.22	7.16±0.44	2.01±0.19	0.78±0.09	4.37±0.54	1.07±0.19	2.95±0.12	1.88±0.18
	JZ2	11.23±0.70	7.28±0.64	2.23±0.29	0.87±0.10	3.62±0.35	1.06±0.11	3.15±0.52	1.85±0.11
	JZ3	11.30±0.87	6.14±0.50	2.41±0.35	0.86±0.10	3.91±0.18	1.14±0.13	2.70±0.22	1.99±0.13
	JZ4	10.91±0.75	6.70±0.50	1.94±0.17	0.84±0.05	4.08±0.42	1.10±0.17	3.00±0.18	1.87±0.23
	JZ5	11.35±0.51	6.89±0.52	1.89±0.38	0.78±0.09	4.52±0.43	1.19±0.16	2.9±0.21	1.85±0.15
	Average	11.52±0.75	7.19±1.12	2.16±0.33	0.83±0.09	4.16±0.50	1.12±0.15	3.04±0.38	1.92±0.17
Male	CS	11.57±0.76	7.22±0.18	2.93±0.23	0.86±0.07	2.72±0.49	0.76±0.06	3.68±0.29	2.01±0.74
	JZ1	11.51±0.71	7.26±0.49	3.15±0.28	0.91±0.11	2.83±0.53	0.70±0.1	2.88±0.29	1.74±0.35
	JZ2	12.70±0.70	7.37±0.41	2.65±0.26	0.92±0.14	2.39±0.50	0.65±0.07	2.96±0.24	1.89±0.15
	JZ3	11.69±0.43	7.49±0.47	2.67±0.28	0.86±0.12	1.99±0.56	0.54±0.10	3.12±0.30	1.82±0.13
	JZ4	11.54±0.75	7.26±0.76	2.68±0.28	0.89±0.27	2.17±0.42	0.75±0.88	3.10±0.16	1.74±0.17
	JZ5	11.64±0.59	7.54±0.50	2.83±0.25	0.70±0.11	2.06±0.21	0.76±0.06	3.04±0.16	1.72±0.13
	Average	11.77±0.74	7.36±0.47	2.82±0.53	0.86±0.16	2.36±0.53	0.69±0.11	3.13±0.35	1.82±0.34

Note: 20 Flowers of each individual were randomly selected (Units: all in mm).

**Table 3:** Two-way ANOVA results for floral dimorphism of *O*. *delavayi*.

Factors	DF	Value of F							
		Corolla length	Corolla width	Anther length	Anther width	Stigma length	Ovary diameter	Sepal length	Sepal width
Sex	1	1.85	0.54	68.64***	0.91	180.98***	164.90***	0.97	1.92
Site	5	1.68	3.99**	1.99	1.79	2.06.	0.66	7.76***	1.35
Sex: Site	5	3.93**	7.39***	4.45**	0.87	2.20	1.30	1.445	0.218
Residual	115								

Note:

**, P < 0.01;

***, P < 0.001;

., P < 0.1

### Pollen characteristics and male function

**Fig 4:**
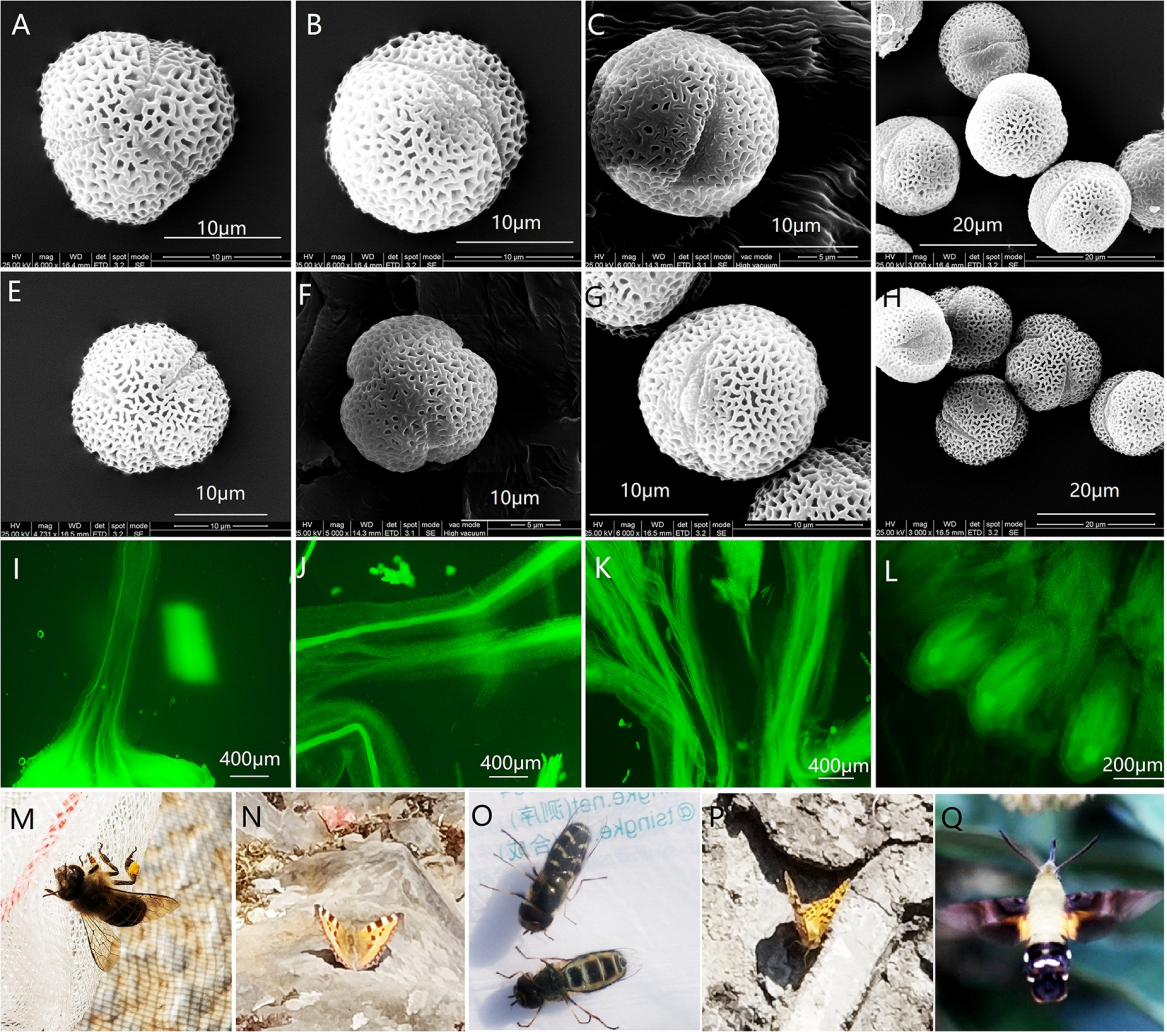
SEM micrographs of pollen characteristics, FM micrographs of pollen tube germination, and pollinators of *O*. *delavayi*. Note: (A-D) Hermaphrodite. (E-H) Male. (I) Pollen tube of self-pollinating hermaphrodite pollen. (J) Pollen tube of outcrossing hermaphrodite pollen. (K) Pollen tube of male pollen. (L) Fertilized ovule. (M-Q) Pollinators: *Apis andreniformis* Smith., *Nymphalis xanthomelas*, *Scaeva pyrastri*, *Argyronome laodice*, *Macroglossum corythus* var. *luteata* (A, E, F) Polar view. (B, C, G) Equatorial view.

In both sexual morphs of *O*. *delavayi*, pollen grains were 3-colpate monad (Fig 4) with 3 germinal furrows distributed symmetrically (Fig 4A–4H). Polar length (R2 = 0.72, P<0.001) ranged from 12.57 μm to 17.83 μm and equatorial diameter (R2 = 0.39, P<0.01) ranged from 15.94 μm to 18.70 μm, both being significantly correlated with sexual morph. Polar views of pollen were mainly circular, while equatorial views were mainly prolate (P/E = 0.77–1.09) (Fig 4), meaning that pollen shapes were spheroid as equatorial diameter was mostly larger than polar length (Table 4). Germinal furrows became narrower from the equator towards the poles (Fig 4B, 4C and 4G). Germinal furrow length ranged from 10.35μm to 15.73μm, and had no correlation with polar axis length, but was positively correlated with sex (R^2^ = 0.84, P<0.001). The apocolpium index (AI) varied from 0.37 to 0.48, and was not significantly different between hermaphrodites and males. Pollen grains of hermaphrodites (P = 15.43–17.83, E = 16.28–18.70) and males (P = 12.57–16.09, E = 15.94–17.39) were both small. In both sexual morphs, equatorial diameter was ca. 1.04–1.1 times larger than polar length. Both polar length (P<0.001) and equatorial diameter (P<0.1) were larger in hermaphrodite pollen grains compared to those in male pollen grains.

**Table 4:** Amount, characteristics, and viability of pollen grains for hermaphrodites and males of *O*. *delavayi* in Yunnan.

	Hermaphrodite	Male	P
Average pollen grains per flower	52479±3097	63663±1767	***
Polar length/μm	16.56±0.70	14.96±0.93	***
Equatorial diameter/μm	17.25±0.77	16.42±0.45	**
P/E ratio	0.96±0.06	0.91±0.06	N/A
Germinal furrow length/μm	14.46±0.85	12.40±0.98	***
Apocolpium index(AI)	0.41	0.40	N.S.
Viability of pollen grains	++	++++	***

Note: units: all in μm except P/E ratio and apocolpium index; means± S.E.; sample size n = 24; P: significance of difference; N/A: not applicable; N.S.: not significant;

*, P < 0.05;

***, P < 0.001;

Y, Yes; “+” represents the degree of viability of pollen grains.

The average number of pollen grains per male flower was significantly greater (1.21 times more) than that of hermaphrodite flowers (F = 73.799, P<0.001). The mean number of pollen grains per male flower was 63,663, while the mean number of pollen grains per hermaphrodite flower was 52,479 (Table 4). The mean P/O ratio was 13,119.8. The pollen of both sexual morphs was fertile, although observation of pollen tubes on the stigma after pollination showed that the germination rate of male pollen was much higher than that of pollen from hermaphrodite flowers through either cross-pollination or self-pollination.

### Stigma receptivity and pollinators

The benzidine-hydrogen peroxide method showed that stigmas of hermaphrodites were receptive during flowering while the stigmas of male flowers were unreceptive. Stigmas of hermaphrodites were already receptive at bud stage, during which period the anthers still had not cracked (Table 5), indicating that the stigmas of hermaphrodite flowers matured before their stamens did.

**Table 5:** Stigma receptivity of male and hermaphrodite flowers of *O*. *delavayi* in Yunnan.

Status of flower	Time of flowering		Stigma receptivity
		male	hermaphrodite
Bud stage	4 days before	—	+
Bud stage	3 days before	—	++
Near-flowering	2 days before	—	+++
Near-flowering	1 day before	—	+++
Early-flowering	1 day	—	++++
Peak-flowering	2 days	—	+++++
Peak-flowering	3 days	—	++++
Peak-flowering	4 days	—	+++
Final-flowering	5 days	—	+
Final-flowering	6 days	—	—

Note: The number of “+” represents the degree of receptivity, “—" means no receptivity.

The pollinators caught include *Macroglossum corythus* var. *luteata* Butler (Fig 4Q), *Nymphalis xanthomelas* Denis et Schiffermüller (Fig 4N), *Argyronome laodice* Pall. (Fig 4P), *Scaeva pyrastri* Linn. (Fig 4O), and *Apis andreniformis* Smith. (Fig 4M). *Scaeva pyrastri* was the most frequent flower visiting insect, visiting once every 8.3 minutes, followed by *A*. *andreniformis*, visiting once every 14.2 minutes. The time when the highest frequency of pollinators visited flowers was 11:00~14:00 daily during peak flowering (Table 6). The average time a pollinator took while visiting a single flower was about 6–8 seconds.

**Table 6:** Pollinators and visiting times during 3 days from 11:00 to 14:00.

Pollinators	visiting times during three days from 11:00~14:00
*Macroglossum corythus* var. *luteata*	3
*Nymphalis xanthomelas*	12
*Argyronome laodice*	8
*Apis andreniformis*	38
*Scaeva pyrastri*	65
Total	126

### Fruit set and gene flow

Under natural conditions, 24 and 48 hours after the pollination experiment, ovule fertilization rate was 43.28% and 51.3, respectively. Fruit set of males artificially outcrossed with hermaphrodites was the highest, with a mean ovule fertilization rate of 63.37% at 24 hours and 76.40% at 48 hours (Table 7). The mean ovule fertilization rate of hermaphrodites artificially outcrossed to other hermaphrodites was also high, with 51.08% at 24 hours and 66.70% at 48 hours (Table 7). When removing stamens from hermaphrodites without any further treatment, mean ovule fertilization rate was about 44.93% at 24 hours and 57.10% at 48 hours. Mean ovule fertilization rate of hermaphrodites that were artificially self-pollinated (flowers artificially pollinated by hand using same pollen and then bagged) was 47.52% at 24 hours and 53.25% at 48 hours, while the same procedure but with no artificial pollination (flowers bagged and stamens not removed) produced a much lower mean ovule fertilization rate of 12.50% at 24 hours and 18.75% at 48 hours. When a gauze was set over hermaphrodite flowers, the mean ovule fertilization rate was 28.13% at 24 hours and 33.93% at 48 hours. Hermaphrodite flowers with stamens removed, sealed in bags and without artificial pollination had a very low mean ovule fertilization rate of 2.05% at 24 hours and 4.17% at 48 hours. Pollination of male stigmas did not produce fruit (Table 7).

**Table 7:** Ovule fertilization rate 24 and 48 hours after artificial pollination.

number	Pollination test	ovule rate after 24h	ovule rate after 48h
1	No picking stamens, no bagging	28.13%	33.93%
2	No picking stamens, bagging	12.50%	18.75%
3	H*H, inbreeding, bagging	47.52%	53.25%
4	H*H, outbreeding, bagging	51.08%	66.70%
5	M*H, bagging	63.37%	76.40%
6	No picking stamens, gauzing	43.28%	51.30%
7	Picking stamens only	44.93%	57.10%
8	Picking stamens, bagging	2.05%	4.17%
9	Pollination of male stigmas	0.00%	0.00%

Note: M = male, H = hermaphrodite; ‘bagging’ refers to sealing flowers in plastic bags; ‘gauzing’ refers to sealing from pollinators but wind pollination is allowed to occur.

A total of 67 alleles were generated using 9 pairs of SSR primers in this study. Each primer had 7.4 alleles on average, and percentage of polymorphic loci on average was 100%. The number of alleles of the 6 populations ranged from 4–10, and the percentage of polymorphic sites was also 100%. When comparing between the 6 populations, JZ4 had the highest values for average alleles (8.000), effective alleles (5.787), observed heterozygosity (0.678), expected heterozygosity (0.766), Shannon’s information index (1.763) and gene flow (0.936), while the *F*_*ST*_ of these values was the smallest. Gene flow between males and hermaphrodites (3.246) was much higher than that between hermaphrodites (1.142). Average alleles (15.222), effective alleles (9.512), expected heterozygosity (0.801), Shannon’s information index (2.205), inbreeding coefficient (0.229) were larger in hermaphrodites than those in males, while the effective alleles (0.617) and genetic differentiation factor (0.180) were smaller than those in males. The genetic differentiation factor among *O*. *delavayi* populations (0.252) was higher than that between males and hermaphrodites (0.202), but gene flow within populations (0.753) was smaller than that between males and hermaphrodites (1.007) (Table 8).

**Table 8:** Genetic variation statistics of different populations and the two sexual morphs.

population	Average alleles *NA*	Effective alleles *Ne*	Observed heterozygosity *Ho*	Expected heterozygosity *He*	Shannon’s information index *I*	Inbreeding coefficient *F*_*IS*_	Genetic differentiation factor *F*_*ST*_	Gene flow *Nm*
CS	7.222	4.993	0.601	0.726	1.636	0.171	0.250	0.750
JZ1	7.222	5.211	0.600	0.720	1.619	0.158	0.263	0.702
JZ2	7.889	5.875	0.678	0.687	1.753	0.020	0.286	0.624
JZ3	6.778	5.350	0.590	0.726	1.623	0.177	0.278	0.755
JZ4	8.009	5.780	0.678	0.766	1.763	0.115	0.211	0.936
JZ5	7.411	5.450	0.638	0.715	1.550	0.121	0.223	0.752
average	7.420	5.443	0.631	0.723	1.657	0.127	0.252	0.753
sex								
Hermaphrodite	15.222	9.512	0.617	0.801	2.205	0.229	0.180	1.142
Male	13.667	7.939	0.658	0.755	2.106	0.127	0.223	0.872
average	14.444	8.726	0.638	0.778	2.156	0.178	0.202	3.246

## Discussion

*O*. *delavayi* can be seen to be clearly morphologically androdioecious as male flowers had well-developed anthers but degraded pistils, whereas hermaphroditic plants had fully developed anthers and pistils. Many morphologically androdioecious species, e.g. in genera such as *Dombeya* Lam. [32] and *Thalictrum* L. [33] have, however, been shown to exhibit cryptic dioecy which led to Charlesworth [5] concluding that the true androdioecious breeding system does not exist. Nevertheless, more recent studies have proven that true functional androdioecy does occur in nature, including studies from several Oleaceae species e.g. *Fraxinus ornus*, *Chionanthus retusus*, *Osmanthus fragrans* [21, 24, 34, 35]. Pollination tests on *O*. *delavayi* provide support that it is functionally androdioecious as male stigmas didn’t set fruit, but pollen was fertile, while hermaphrodite pollen and stigmas were both fertile, i.e. males only show male function, while hermaphrodites show both.

The theoretical evolutionary stable strategy model (ESS model) [5, 36] states that stable androdioecy requires that males sire more than twice as many offspring as hermaphrodites [37], and the ratio of male individuals in functionally androdioecious populations may be lower than 0.5 [38, 39] when the male function has a higher fitness advantage. However, in several members of the olive family (Oleaceae), androdioecy occurs with higher frequencies of males than predicted by theory, which can be explained by their self-incompatibility system [8]. In the 6 populations of *O*. *delavayi* studied, male ratio ranged from 28% to 40% and the ratio decreased with increasing elevation, most likely due to the poor environmental conditions at higher elevations. To cope with the harsher conditions of high elevations, plants often have reduced reproductive allocation, which consequently leads to a decrease of female reproduction [40] and inbreeding becomes more common. The ESS model indicates that male functional distribution decreases with increasing rates of inbreeding [41], while higher fitness is afforded to hermaphrodites. The generally low male ratio and its decrease with elevation is also likely to be due to the high degree of fitness of self-compatible hermaphrodites within the populations. Nevertheless, high male ratios can increase outcrossing and community genetic diversity, contributing to a species long-term fitness.

The fact that male flowers produce significantly larger quantities of smaller, lighter pollen compared to hermaphrodites, that likely facilitate their transmission by wind, has likely contributed to the maintenance of androdioecy in *O*. *delavayi*. Furthermore, pollen germination capacity of male pollen was about twice that of the hermaphrodite pollen (Fig 4, Table 5). Shape of pollen, and size and attractiveness of flowers, were ruled out as possible factors influencing the breeding system as pollen grains of male and hermaphrodite flowers differed very little in shape and males and hermaphrodites had similar flower sizes that were equally attractive to pollinators. Thus, greater pollen transmission and germination capacity is thought to be significant for the ambophily (wind and insect pollination) strategy and ultimately for the maintenance of androdioecy [20] in the 6 populations of *O*. *delavayi*.

Mean P/O was 13,119.8, implying that the mating system of *O*. *delavayi* is specialized outcrossing [29]. Study of floral structure found that anthers were positioned higher than stigmas (Fig 1D–1F), mean solitary flower diameter was more than 6 mm (Table 2), and it was also clear that the female stigmas matured earlier than the anthers did. Owing to these characteristics, *O*. *delavay*i has an OCI index of 5. According to the criteria of Dafni [28], the breeding system of *O*. *delavayi* can be seen as outcrossing, partially self-interactive, and requiring pollinators.

Pollination tests found that males and hermaphrodites both had fertile pollen grains, providing further proof that the mating system of *O*. *delavayi* was mainly outcrossing. This was shown by how a) pollination between males and hermaphrodites and pollination between just hermaphrodites both produced fruit, with the ovule fertilization rate of the former being higher (77.6%) than that between just hermaphrodites (66.7%) as well as that under natural conditions (33.9%); b) pollen on male stigmas could not germinate at all; c) the ovule fertilization rate of completely self-pollinating hermaphrodites was lower (18.8%) than that of setting gauze to prevent pollinating insects (51.3%); d) the ovule fertilization rate of hermaphrodite flowers with only stamens removed was 57.1%. Self-pollination could produce fruit, but there was a certain degree of self-incompatibility which caused a reduction in fitness of self-compatible hermaphrodites relative to males, while also contributing to the maintenance of the males two-fold fitness advantage [5, 36], supporting the view that self-incompatibility (SI) systems and separate sexes evolve together [42]. Our pollination survey also indicated that besides wind, insects may also play an important role in pollination. This can be seen by how the pollinator monitoring experiments found that a significant number of insects visited *O*. *delavayi* flowers (Table 6).

Gene flow between males and hermaphrodites was much higher (3.246) than gene flow between the six populations (0.753) and gene flow between hermaphrodites (1.142), indicating that there was much outbreeding between males and hermaphrodites, while the gene flow between populations was very weak [43, 44]. This provides further support for the view that the breeding system of *O*. *delavayi* is functional androdioecy. The *F*_*ST*_ between populations (0.252) was higher than that between males and hermaphrodites (0.202), meaning that the genetic differentiation between populations accounts for 25.2%, and the genetic differentiation within populations accounts for 74.8%. This also provides evidence that *O*. *delavayi* is an out-breeding plant. The low genetic differentiation factor between males and hermaphrodites indicates the stability of the *O*. *delavayi* breeding system. The Shannon’s information index also shows that there is high genetic diversity both across the 6 populations and also in the two sexual morphs.

All of this evidence allows us to conclude that *O*. *delavayi* is indeed a functionally androdioecious plant. It has been suggested that androdioecy may have originated from dioecious progenitors, with research on certain herbaceous plants corroborating this theory [45–47]. However, for woody androdioecious plants, especially in the family Oleaceae, several species have already been shown to be intermediate stages along an evolutionary pathway from hermaphroditism to dioecy [3, 7, 13, 19, 20]. Certain limitations in the life-history traits of woody plants favor the transition of their breeding system from hermaphroditism to androdioecy, such as the relatively long period before flowering and fruiting [48]. Males have been found to more readily allocate resources to asexual reproduction and vegetative growth, such as through root suckers [9, 49–50] and, as such, can have higher fitness in harsh environments. The lower fitness of hermaphrodites under harsh conditions is hypothesized to have led to the formation of male mutants with their reduced reproductive input affording them higher adaptability in adverse environments [5, 20, 36, 51]. These mutants are hypothesized to have been preserved in harsh environments, leading to the formation of androdioecious populations.

In our study, the simple flowers with small and open corollas [52] and large amount of small pollen produced, combined with the breeding system of androdioecy, artificial pollination results, OCI index and P/O, shows that *O*. *delavayi* is ambophilous (wind and insect pollinated). The development from hermaphroditism to dioecy through androdioecy linked to ambophily is one of the three possible evolutionary pathways that lead to anemophily (wind pollination) [20], with ambophily being an intermediate state in the transition from biotic pollination to anemophily. Hermaphrodites are more suitable for biotic pollination (zoophily), which is more ancestral than ambophily in angiosperms [20, 53], while simple anemophilous flowers are considered to be more advanced. This also provides further proof that androdioecy is the intermediate state in the evolutionary pathway from hermaphroditism to dioecy in *O*. *delavayi*. High outcrossing rates, as found in *O*. *delavayi*, are necessary for androdioecy to evolve from hermaphroditism [15]. In addition, characteristics of the degraded pistil with unhealed carpel of male flowers provide further evidence that males may have evolved from hermaphrodites. Conversely, if female flowers of dioecious plants developed stamens to improve their pollination guarantee, male plants should not have degenerate pistils present in the flowers, and hermaphrodites would also not be self-incompatible.

## Conclusion

We provide strong evidence that *O*. *delavayi* is functionally androdioecious and speculate that this breeding system is the intermediate state in the evolutionary pathway from hermaphrodism to dioecy through the pistil degenerating to form staminate flowers that are stably preserved. This very rare breeding system found in the endemic *O*. *delavayi* highlights that this species should be allocated greater conservation concern as its germplasm will be fundamental for further research on the evolution of breeding systems in flowering plants. As the area occupied by the species can be considered very small with natural regeneration indicated to be slow, with very few seedlings present on the forest floor, measures need to be taken to conserve the species, such as establishing protected areas. Our findings that the fruit setting rate under natural conditions was relatively low, while artificial pollination significantly increased the fruit set, implies that manually assisting pollination could be an effective conservation measure for increasing fruit set and further establishment of this rare species.
